# P-1390. Anorectal Human Papillomavirus (HPV) Infection in HIV-Positive Men who have Sex with Men (MSM) in Nicaragua

**DOI:** 10.1093/ofid/ofae631.1566

**Published:** 2025-01-29

**Authors:** Miguel A Meléndez-Avendaño, Sunaya Marenco-Avilés, Kevin Gavarrete-Rivas, Guillermo D Porras-Cortés

**Affiliations:** Hospital Dr. Fernando Vélez Paiz, Managua, Managua, Nicaragua; Hospital Dr. Fernando Vélez Paiz, Managua, Managua, Nicaragua; Hospital Dr. Fernando Vélez Paiz, Managua, Managua, Nicaragua; Hospital Dr. Fernando Vélez Paiz, Managua, Managua, Nicaragua

## Abstract

**Background:**

HIV-positive men who have sex with men (MSM) are at increased risk for sexually transmitted infections, including anorectal HPV, with the consequent likelihood of neoplasia at that level. There is a complete lack of information on this situation in Nicaragua. This study explores and establishes for the first time the prevalence of anorectal HPV infection in HIV-positive MSM and its correlation with cytological abnormalities, and some associated behavioral, virological, and immunological factors in Nicaragua.

**Figure 1.**
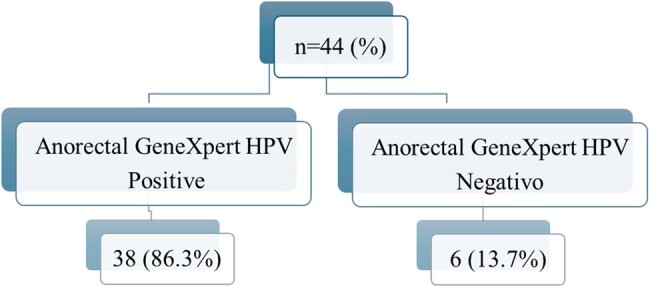
Prevalence of HPV Anorectal Infection in MSM HIV Positive Diagnosed by RT-PCR Xpert HPV.

**Methods:**

A prospective, multicenter cohort study was conducted between January 2023 at December 2023, at Dr. Fernando Vélez Paiz Hospital, Antonio Lenin Fonseca Hospital, and Manolo Morales Hospital in the city of Managua, Nicaragua. HIV-positive MSM patients were enrolled to the study with prior consent. Anorectal swab samples were processed by molecular method for HPV detection (GeneXpert HPV®) of different genotypes individually or grouped into genetic patterns (16, 18/45, P3: 31/32/35/52, P4: 51/59, and P5: 39/56/66/68). In addition, anorectal Pap smears were performed for cytopathology study. All samples were processed in the microbiology and pathology laboratories of the Dr. Fernando Vélez Paiz Hospital, the main center of the research.

**Figure 2.**
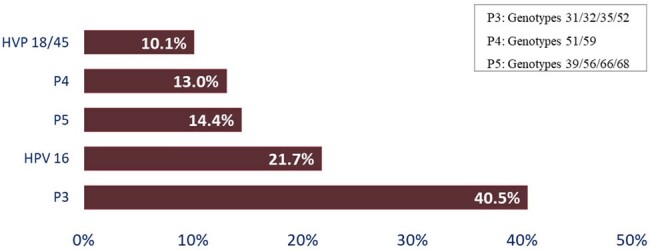
Different Genotypes Detected by Xpert HPV Anorectal Infection in MSM HIV Positive.

**Results:**

A total of 44 patients were enrolled in the study. A significant proportion of patients declined to participate in the study. The mean age was 35.2 ± 12.4 years. Most of the patients reported having a passive or versatile sexual role. 22.7% were naïve to antiretroviral therapy (ART). The prevalence of anorectal HPV infection was 86.3% (Figure 1). The most frequent genotype was HPV 16 (21.7%) (Figure 2). In the combined genotype patterns, P3 (31/32/35/52) was observed in 40.5% of patients. All patients were infected with more than one genotype. 31.5% of those infected had cytological abnormalities, the most frequent being low-grade squamous intraepithelial lesion (Table 1). HPV-positive patients were younger, had a higher viral load, and a lower CD4+ lymphocyte count (Table 2).

**Table 1.**
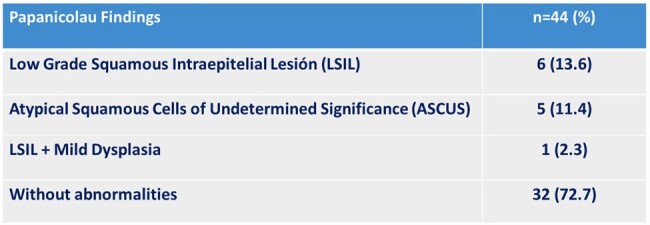
Abnormalities in Anal Papanicolau in MSM HIV-positive con HPV Infection Diagnosed by Xpert HPV.

**Conclusion:**

The prevalence of anorectal HPV infection in this study is high, with serotype 16 being the most frequent. Almost a third of them have altered cytology, with low-grade squamous intraepithelial lesion being the most frequent finding.

**Table 2.**
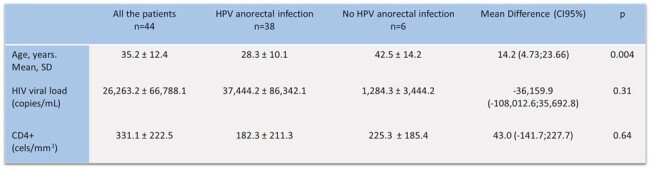
Differences in Age, HIV Viral Load and Count of Lymphocyte CD4+ in MSM HIV-Positive with and wihtout HPV Infection

**Disclosures:**

**All Authors**: No reported disclosures

